# Effectiveness and micro-costing of the KiVa school-based bullying prevention programme in Wales: study protocol for a pragmatic definitive parallel group cluster randomised controlled trial

**DOI:** 10.1186/s12889-016-2746-1

**Published:** 2016-02-01

**Authors:** Suzy Clarkson, Nick Axford, Vashti Berry, Rhiannon Tudor Edwards, Gretchen Bjornstad, Zoe Wrigley, Joanna Charles, Zoe Hoare, Obioha C. Ukoumunne, Justin Matthews, Judy Hutchings

**Affiliations:** 1 Centre for Evidence Based Early Intervention, School of Psychology, College of Health and Behavioural Sciences, Bangor University, Nantlle Building, Normal Site, Bangor, Gwynedd LL57 2PX Wales, United Kingdom; 2 Dartington Social Research Unit, Lower Hood Barn, Dartington, TQ9 6AB Devon United Kingdom; 3 NIHR CLAHRC South West Peninsula (PenCLAHRC), University of Exeter, Exeter, Devon EX1 2 LU United Kingdom; 4 Centre for Health Economics and Medicines Evaluation, Bangor University, Ardudwy Hall, Bangor, Gwynedd LL57 2PZ Wales, United Kingdom; 5 NWORTH CTU - North Wales Organisation for Randomised Trials in Health, Bangor University, Y Wern, Normal Site, Holyhead Road, Bangor, Gwynedd LL57 2PZ Wales, United Kingdom

**Keywords:** Bullying, Victimisation, Emotional well-being, Absenteeism, School-based, Intervention, Micro-costing, Randomised, KiVa, Prevention

## Abstract

**Background:**

Bullying refers to verbal, physical or psychological aggression repeated over time that is intended to cause harm or distress to the victims who are unable to defend themselves. It is a key public health priority owing to its widespread prevalence in schools and harmful short- and long-term effects on victims’ well-being. There is a need to strengthen the evidence base by testing innovative approaches to preventing bullying. KiVa is a school-based bullying prevention programme with universal and indicated elements and an emphasis on changing bystander behaviour. It achieved promising results in a large trial in Finland, and now requires testing in other countries. This paper describes the protocol for a cluster randomised controlled trial (RCT) of KiVa in Wales.

**Methods/Design:**

The study uses a two-arm waitlist control pragmatic definitive parallel group cluster RCT design with an embedded process evaluation and calculation of unit cost. Participating schools will be randomised a using a 1:1 ratio to KiVa plus usual provision (intervention group) or usual provision only (control group). The trial has one primary outcome, child self-reported victimisation from bullying, dichotomised as ‘victimised’ (bullied at least twice a month in the last couple of months) versus ‘not victimised’. Secondary outcomes are: bullying perpetration; aspects of child social and emotional well-being (including emotional problems, conduct, peer relations, prosocial behaviour); and school attendance. Follow-up is at 12 months post-baseline. Implementation fidelity is measured through teacher-completed lesson records and independent school-wide observation. A micro-costing analysis will determine the costs of implementing KiVa, including recurrent and non-recurrent unit costs. Factors related to the scalability of the programme will be examined in interviews with head teachers and focus groups with key stakeholders in the implementation of school-based bullying interventions.

**Discussion:**

The results from this trial will provide evidence on whether the KiVa programme is transportable from Finland to Wales in terms of effectiveness and implementation. It will provide information about the costs of delivery and generate insights into factors related to the scalability of the programme.

**Trial registration:**

Current Controlled Trials ISRCTN23999021 Date 10-6-13

## Background

There is now general consensus among researchers that bullying is defined as verbal, physical or psychological aggression repeated over time that is intended to cause harm or distress to the victims who are unable to defend themselves [1]. Bullying is a major international social, physical and mental health concern owing to its effects and prevalence [2, 3].

Victimisation, or being bullied, carries numerous detrimental and long-term consequences, including depression [4, 5], psychological maladjustment [4, 6–8], high-risk health behaviours, such as drinking, smoking and substance abuse [9–11] and suicidal ideation and suicide [4, 12]. Victimisation has also been associated with increased school absence [13], poorer educational attainment [14, 15] and lower lifetime earnings [16, 17].

Bullying is widespread and occurs regularly in most school settings [18], with many children frequently observing some form of bullying at school [19, 20]. A World Health Organization study, involving over 200,000 children aged 11 to 15 years from 39 countries, reported that one in 10 children worldwide say that they are bullied (bullied at least two or three times in the past couple of months [21]. A more recent survey, involving over 580,000 children aged 11, 13 and 15 years from 33 countries (31 European and two North American), reported that 29 % of children were ‘occasional victims’ (bullied at school once in the past couple of months) and 11 % were ‘chronic victims’ (bullied at least two or three times in the past couple of months) [22]. In at least 85 % of bullying incidents, peers are reported to be present [23]. Teachers report bullying incidents far less frequently than pupils [24] and also often perceive the incidents to be less severe [24, 25], emphasising the need for teacher awareness training and a clear school definition of bullying.

The focus of anti-bullying interventions in schools has altered over the last two decades. Earlier interventions were typically focused on the individual or a small target group, and involved providing therapy and counselling and seeking to enhance children’s social competence [26, 27]. More recently, a more encompassing multi-faceted approach has been developed that involves not only the bully and the victim but also seeks to change the social dynamics within the peer group and the wider school community. Targeted interventions concentrated solely at the level of the bully and/or the victim have had little success in reducing bullying [27, 28] whereas multiple level whole-school approaches have demonstrated significant effectiveness in reducing bullying behaviour [27, 29]. An extensive systematic review of school-based bullying prevention programmes identified 53 evaluations (reported on in 89 publications) [29]. Of these, 44 contained sufficient detail to calculate effect sizes and were included in meta-analyses, which found that, on average, bullying decreased in relative terms by between 20 % and 23 % and victimisation by between 17 % and 20 %. The individual components of each programme were identified based on published papers and private communications with programme evaluators. The analysis identified that, in order of importance, the three most important programme elements associated with a decrease in bullying perpetration were parent training/meetings, improved supervision in playgrounds, and (higher) programme intensity for children (measured in terms of number of hours). The three most important elements associated with reduced victimisation were the formal engagement of peers in tackling bullying, firm (‘punitive’) disciplinary methods and parent training/meetings. The duration and intensity of the programme for both teachers and children were significantly associated with a decrease in both victimsation and bullying. There is little information available about the costs or cost-effectiveness of anti-bullying programmes [17, 30].

This paper describes the protocol for a pragmatic cluster RCT of the KiVa bullying prevention programme in Wales. KiVa is an acronym for ‘Kiusaamista Vastaan’ which, translated, means ‘against bullying’ and also ‘kiva’ is a Finnish adjective for ‘nice’. KiVa is an evidence-based programme developed in Finland for children aged 7 to 15 years. In 2006, the Finnish Ministry of Education and Culture contracted Professor Salmivalli at Turku University to develop and evaluate a school based anti-bullying programme. This was in recognition that legislative requirements in Finland over the decade prior to 2006 that schools should have a bullying prevention policy had resulted in no change to bullying prevalence figures.

The KiVa programme includes universal actions, directed at the class and school level, and indicated actions, for addressing incidents of bullying. It offers an innovative approach to bullying in that it focuses on the role of bystanders (fellow pupils who witness bullying events). Through class lessons, it teaches children to recognise what is, and is not, bullying and how to respond when they see bullying. Lessons are grouped into three units aimed at children aged 6–9, 10–12 and 13–14 respectively. This approach is based on extensive research [31] showing that victims report distress when others do nothing to help and that bullies tend to behave aggressively to attain higher status and are reinforced by onlookers’ apathy or encouragement. In addition, this research found that when bystanders do intervene, the bullying tends to stop.

A RCT of KiVa in Finland involving more than 8,000 children aged 10–12 years in 78 schools found that it was effective for reducing self-reported victimisation (effect size (Cohen’s d) = 0.17) and bullying perpetration (d = 0.10) [32]. The effects were seen across all types of bullying, including verbal, physical, racist, sexual and cyber-bullying [33]. Following the success of this trial, the Finnish government supported a national roll-out of KiVa and it is now delivered in over 90 % of schools in Finland (pupils aged 7 to 15 years; approximately 2,700 schools). A non-randomised evaluation of this roll-out has also demonstrated positive effects, albeit smaller in size than in the trial [34].

This trial in Wales is one of several evaluations of KiVa currently being undertaken in Europe (others are taking place in Estonia, Italy and the Netherlands). The Welsh education system today is in a similar position to that of the Finnish education system prior to 2006 in that the Welsh Government has relied on legislative change and guidance issued to schools to reduce bullying. Section 175 of the *Education Act* 2002 [35] places a duty on local education authorities (LEAs) and governing bodies of maintained schools to safeguard and promote the wellbeing of all pupils, which includes a responsibility to tackle bullying in all forms [36]. Schools are required to have an anti-bullying policy that sets out procedures for recording bullying incidents, investigating and dealing with incidents, supporting victims and disciplining bullies [37]. According to the first comprehensive national survey in Wales of the prevalence and incidence of school bullying, conducted in 2010, approximately 32 % of Year 6 pupils (aged 10–11) reported that they had been bullied in the last two months, rising to 47 % in the last year [38].

A small opportunistic pre-post pilot study of KiVa was conducted in Wales in the academic year 2012–2013, with 17 schools using Unit 2 of the programme (the first to be translated into English) [39]. The pilot measured levels of self-reported victimisation and bullying before and after nine months (one academic year) of implementation of KiVa. It found statistically significant reductions in self-reported victimisation (16 % to 9 %) and bullying (6 % to 2 %) [40].

The cluster RCT described in this paper will be conducted throughout Key Stage 2, with pupils aged 7 to 11 years, and using Units 1 and 2 (Unit 1 has been translated into English since the pilot study was conducted). The study aims to test the effectiveness of KiVa in Wales, measure the fidelity of its implementation, and examine factors predicted to affect the scalability of the programme. The results will indicate the extent to which the programme is ‘transportable’, that is, whether it is as effective in Wales as in Finland. The results of the trial will be of interest to the international child behaviour policy and practice community, and also to policy makers and commissioners in Wales where the education inspection service has identified tackling bullying in schools as a priority [36]. Should the results be promising, the insights gained through implementing KiVa will be used to develop plans for a model of the programme that could be scaled up to all primary schools in Wales.

## Methods/design

### Design

The study is a two-arm waitlist control pragmatic definitive parallel group cluster randomised controlled trial with a 1:1 allocation ratio. Participants will be recruited at the end of the 2012/13 academic year, with outcomes measured at the end of the 2013/14 academic year.

### Study setting

The setting is mainstream state-maintained primary and junior schools in Wales. Primary schools serve children aged 4 to 11 years and junior schools serve children aged 7 to 11 years.

### Participants

The study will recruit pupils in Years 2, 3, 4 and 5 in the participating schools at the end of the 2012/13 academic year.

Headteachers and teachers in participating schools will be able to review the questionnaires for children and determine whether they are suitable for children with learning difficulties. Schools that cater exclusively for children with special needs will not be invited to participate. This is because at the time of designing the study there was no evidence from Finland of the effectiveness of the programme with pupils in such settings.

### Recruitment and retention

The study will recruit schools through two half-day conferences in South Wales and North Wales respectively (March 2013). The conferences will provide information on: the KiVa programme and prior research on its effectiveness; the training, implementation and support that will be provided; and the nature of the proposed evaluation. Participation will be offered on a first-come-first-served basis to those schools that confirm, in writing, their commitment to (a) delivering the curriculum to all Key Stage 2 pupils and (b) participating in the evaluation. School recruitment will be completed by the end of April 2013.

The incentives for school participation are free school materials and training and KiVa registration for two years (the intervention schools will be able to implement KiVa for a further year beyond the trial and the wait list control schools will also get to implement KiVa for two years post trial). There are no adverse consequences (e.g., loss of resources or money, or negative publicity) for schools of discontinuing the intervention or deviating from the protocol. The proportion of children leaving schools or being absent at the time of the follow-up assessment is unlikely to be more than 10 %.

### Sample size

We will randomise 10 schools (clusters) to each of the intervention and control arms (20 schools altogether) and recruit all children from Years 2 to 5, following them up when in Years 3 to 6. Assuming there are 1.25 classes in each year group, and 25 children per class on average, there should be 125 potentially eligible children in each school. Based on a consent rate of 95 % and a drop-out rate of 10 % we anticipate that 1070 children will provide follow-up data in each trial arm at 12 months post-baseline (2140 children altogether). The percentage of victimised children, the primary outcome, is estimated to be 16 % [40]. Taking into account an assumed intra-cluster (intra-school) correlation coefficient of 0.025 [29], our sample size will be large enough to detect a halving from 16 % to 8 % in the percentage of victimised children with just over 80 % power (81.6 %) at the 5 % (2-sided) level of significance.

### Randomisation

As KiVa is a whole school intervention, the 20 schools (clusters) will be randomly allocated on a 1:1 basis to intervention and control conditions. Randomisation will be carried out by an independent registered trials unit at Bangor University (the North Wales Organisation for Randomised Trials (NWORTH)). Complete list randomisation using the dynamic adaptive algorithm^1^ [41] will be implemented, by a validated computer package with stratification by size of school (large/small split by the median) and proportion of children eligible for free school meals (high/low split by the median). Researchers are unable to remain blind to school allocation, as the implementation evaluation will be undertaken with schools when they are delivering the programme. However, the statisticians on the trial will be blind to allocation status. Schools will be informed of their assignment (intervention or control group) by Bangor University towards the end of May 2013.

### Intervention

The duration of the KiVa programme is one full academic year. As described above, the intervention contains universal and indicated elements. The components aim to affect norms, skills, behaviour, attitudes, and classroom and school climate. KiVa provides training, resources, class lessons, online activities and advice and support for parents that are based on the internationally agreed and research-based definition of bullying. Within the universal element there are three curriculum units, for children aged 6 to 9 (Unit 1), 10 to 12 (Unit 2) and 13 to 14 (Unit 3). Units 1 and 2 will be used in the Wales trial. Each unit contains 10 × 90-min lessons to be delivered monthly. Lessons include film clips, group discussions and exercises. In Wales, lessons are usually delivered as half lessons – i.e., 20 × 45-min lessons, delivered approximately fortnightly. The Key Stage 2 KiVa programme maps onto the Welsh Personal and Social Education (PSE) curriculum and covers over 50 % of it. A copy of the PSE curriculum/KiVa mapping will be provided to the intervention schools to enable them to incorporate the KiVa lessons into their school PSE plan. Additional universal elements are online games (which can also be played at home), school-wide posters and high-visibility vests for staff to wear in the playground during breaks to remind children they are in a KiVa school.

KiVa provides a standard protocol for teachers to address confirmed cases of bullying (the indicated element). Members of the KiVa team meet with the bullied victim and perpetrator(s) separately. If there is more than one perpetrator, the KiVa team will hold an additional meeting with each of the perpetrators. The discussion with the perpetrator can be approached in either a ‘confrontational’ or ‘non-confrontational’ manner. In the confrontational approach, the teacher refers to the perpetrator’s role in the bullying incident explicitly, before asking them to agree to a plan to address the problem. In the non-confrontational approach, the teacher simply explains that the victim is having a difficult time and asks the perpetrator to commit to ways to help them feel better. High-status peers are encouraged to befriend and support the victim and to work with the teacher to think of ways to do this. A follow-up discussion with both the victim and the bully (or bullies) is scheduled for two weeks later to establish whether the bullying behaviours have stopped, and, if necessary, to repeat the process or ultimately to move to other sanctions.

Support and feedback sessions and a helpline will be provided to assist with any queries and improve school adherence to the intervention protocol. Intervention delivery begins at the start of the school year (September).

Intervention training will be provided in the summer term prior to the academic year in which implementation starts (i.e., June/July 2013 for intervention schools). Accredited KiVa trainers (members of the Finnish KiVa team, JH and SC) will provide training in Bangor and Cardiff respectively. Two members of the teaching/management team from each of the schools will be required to attend the two-day training. Follow-up twilight school-based training sessions will be delivered to all school staff (JH and SC). All Key Stage 2 regular class teachers will then deliver the KiVa curriculum to pupils.

### Control

Control schools will provide services as usual. PSE is an essential element of the basic curriculum for all pupils at maintained schools in Wales [42]. The PSE curriculum aims to develop and explore pupils’ values and attitudes, equip them to live safe and healthy lives, promote self-respect, celebrate diversity, and empower participation in school and community life as responsible citizens. The PSE Framework states that “it is the responsibility of the school to plan and deliver a broad, balanced programme of PSE to meet the specific need of the learners” (p.3). Control schools will continue to use their existing plan for covering the PSE curriculum. Schools use various strategies to improve social interactions, such as peer support/mentoring schemes (62 % of UK schools [43]), and to prevent or address bullying. No other programmes or strategies will be prohibited during the trial, so that there is no interfererence with standard school practice. The trial uses a waitlist control design and KiVa will be implemented in the control schools after the end of the trial.

### Outcome measures

The effectiveness objectives of the trial are to evaluate whether KiVa:

1. reduces pupil-reported victimisation (primary outcome) and bullying perpetration, as measured by the KiVa pupil online survey [32];
2. improves children’s emotional well-being as measured by the emotional difficulties subscale of the teacher-completed Strengths and Difficulties Questionnaire (SDQ) [44, 45];
3. has a positive impact on other aspects of children’s social and emotional well-being, as measured by the subscales of conduct problems, peer relations and prosocial behaviour, the “total difficulties” score and the impact score on the teacher-completed Strengths and Difficulties Questionnaire (SDQ) [44, 45] (see below for further details);
4. reduces school absenteeism as measured by school administrative data on authorised and unauthorised half-day absences.

#### Victimisation and bullying

The primary study outcome is pupil self-reported victimisation, occurring at least twice a month. Both victimisation and one of the secondary outcomes, pupil self-reported bullying perpetration, will be measured using the Bully/Victim Questionnaire (BVQ) [46], which is part of the KiVa pupil online survey [32], completed by the study participants. The global items: “How often have you been bullied at school in the last couple of months?” and “How often have you bullied others at school in the last few months?” will be used to measure victimisation and bullying, respectively. Pupils respond to both items on a five-point scale (0 = “not at all”, 1 = “once or twice”, 2 = “2 or 3 times a month”, 3 = “about once a week”, 4 = “several times a week”). Each item will be dichotomised for analysis so that those scoring 2 to 4 will be classified as victimised/bullied others and those scoring 0 to 1 as not victimised/did not bully others. This categorisation is conceptual (bullying concerns *repeated* acts), but it is supported by empirical research showing that there are large and highly significant differences between these groups on internalising problems (for victims) and externalising problems (for bullies) [47].

#### Social and emotional well-being

In order to measure aspects of children’s social and emotional well-being (also secondary outcomes), the teacher-reported Strengths and Difficulties Questionnaire (SDQ) [44, 45] will be administered at baseline and at 12-month follow-up. It is a 25-item screening measure widely used in developmental, social, clinical and educational studies to measure children’s mental health and well-being. The teacher version can be completed for children aged 4 to 17 years. It comprises five subscales (each with 5 items) assessing hyperactivity, conduct, emotional difficulties, peer relations and pro-social behaviour, respectively, over the past six months. There are three response options for each item (0 = “not true”, 1 = “somewhat true”, 2 = “certainly true”). For each of the subscales the score can range from 0 to 10; a higher score indicates more problems for all subscales apart from the prosocial subscale, for which a higher score indicates more prosocial behaviour. The “total difficulties score” is calculated by summing the 20 items that comprise the first four subscales listed above (total score ranges from a possible 0 to 40, with higher scores indicating greater problems).

The SDQ also has a brief ‘Impact supplement’ which starts with a single question about whether the child has difficulties with emotions, concentration, behaviour, or being able to get on with other people (response set: “No”, “Yes – minor difficulties”, “Yes – definite difficulties”, and “Yes – severe difficulties”). If the answer is “Yes” there are four additional questions, focusing respectively (in the teacher version) on: chronicity, or duration (response set: “less than a month”, “1–5 months”, “6–12 months”, “over a year”); distress to the child (response set: “not at all”, “only a little”, “quite a lot”, “a great deal”); impact on the child’s everyday life in terms of peer relations and classroom learning respectively (response set: 0 = “not at all”, 0 = “only a little”, 1 = “quite a lot”, 2 = “a great deal”); and burden to the teacher or class as a whole (response set: 0 = “not at all”, 0 = “only a little”, 1 = “quite a lot”, 2 = “a great deal”). The teacher-report impact score is calculated by summing responses to three items, namely (i) whether the difficulties upset or distress the child, and impact on (ii) peer relations and (iii) classroom learning, with the total score ranging from 0 to 6, where higher scores indicate greater impact.

A review of the psychometric properties of the teacher-completed SDQ, examining 26 studies involving teachers of children aged between four and 12 years, estimated the overall Cronbach’s alpha of inter-item reliability to be 0.73 for the emotional symptoms subscale, 0.82 for prosocial behaviour, 0.70 for conduct problems, 0.63 for peer problems, 0.82 for the total difficulties score and 0.85 for the impact score.^2^ The pooled test-retest reliability correlation from six studies was also high for the total difficulties score (Pearson’s correlation (*r*) =0.84) and the impact score (*r* = 0.68) [48].

#### School absenteeism

School records of authorised and unauthorised half-day absences will be provided at the pupil level by school administration staff for participating pupils in the study for the academic years 2012–2013 (baseline) and 2013–2014 (12-month follow-up). These data are routinely collected by schools for all pupils as a legal requirement. Schools will provide the anonymised attendance data linked to the KiVa IDs to ensure that pupil anonymity is protected.

### Data collection

Table 1 summarises when the outcome data will be collected. Baseline data will be collected via pupil and teacher surveys in intervention and control schools in June/July 2013 for children in Years 2, 3, 4 and 5 (i.e., about to enter the Key Stage 2 Years 3, 4, 5, and 6). Data using the same measures will be collected at 12 months post-baseline (June/July 2014) for children coming to the end of Years 3, 4, 5, and 6.^3^Ethnicity, free school meals and SEN status (for baseline, or point of entry to school if the pupil joined the school subsequently) and absence data (for the academic years 2012–2013 and 2013–2014) will be collected by Autumn 2015.

**Table 1:** Timing of data collection and intervention delivery

	June/July 2013	Sept 2013–July 2014	June/July 2014	By Autumn 2015
Intervention arm schools	Baseline outcome data collected for Years 2, 3, 4, 5	Delivery of KiVa programme	12-month post-baseline outcome data collected for Years 3, 4, 5, 6	Collection of school administrative data on ethnicity, free school meals, SEN status and absence.
Control arm schools	As above	Provision as usual	As above	As above

### Analysis of effectiveness of the KiVa intervention

The analysis will estimate differences at 12-month follow-up between the two trial arms, adjusting for baseline data. Statistical analyses will be reported in accordance with the CONSORT guidelines for cluster RCTs [49].

Baseline characteristics of the schools and pupils will be summarised separately for each trial arm using means and standard deviations (or medians and interquartile ranges) for continuous variables and numbers and percentages for categorical variables.

Comparison of outcomes at follow-up will be based on the intention-to-treat (ITT) principle with schools (clusters) and pupils analysed according to the trial arm they were allocated to, irrespective of the level of intervention actually received. The main reported findings will be based on analyses of 20 multiply imputed datasets generated (each using 10 cycles) using the fully conditional (“chained equations”) approach to “fill in” missing values [50, 51]. All study outcomes (primary and secondary), trial arm status, stratification and prognostic factors/confounders (outcome score at baseline, age, gender, ethnicity, qualifying for free school meals (child level) and special education needs (child level)) and potential effect modifiers of interest (see below) will be included in the imputation model.

Binary outcomes will be compared between trial arms using marginal logistic regression models using Generalised Estimating Equations with information sandwich (“robust”) estimates of standard error assuming an exchangeable correlation structure. Continuous outcomes will be compared using random effects linear regression. Both methods allow for correlation of outcomes within schools (clusters).

Tests of interaction will be performed to investigate whether the intervention effect on victimisation status at follow-up is moderated by victimisation status at baseline, gender and age, and whether the intervention effect on bullying status at follow-up is moderated by bullying status at baseline, gender and age. Tests of interaction will be considered to provide significant results if the p-value is less than 0.05. These analyses are purely exploratory, with any significant findings needing to be replicated in other studies to give them credence. Furthermore, we acknowledge the low power of these analyses.

The findings from analyses of imputed data will be contrasted with sensitivity analyses based on analysis of complete cases only (listwise deletion). The amount of missing data will be reported as a percentage for the main outcomes in each trial arm along with the amount of data recovered in the imputation analysis. The characteristics of children lost to follow-up will be compared to those retained in each trial arm to assess the nature of attrition.

Stata 13.1 will be used for the analyses using the *mi impute* and *mi estimate* commands to generate imputed datasets and analyse these, respectively.

### Process evaluation

#### Implementation fidelity

The objective of the first part of the process evaluation is to describe the level of implementation fidelity as assessed by the teacher-completed online Teacher Lesson Record Books (for class lessons) and independent observations (for the school-wide element). Data on the fidelity of programme implementation will be collected for intervention arm schools and waitlist control schools when they subsequently deliver the intervention. Although the analyses of effectiveness are based on data collected during the trial period only, the process evaluation will use data from the waitlist controls as well.

Quantitative data relating to the delivery of the KiVa lessons will be collected using KiVa online teacher lesson record books. The teachers document the following: time spent preparing each lesson; time spent delivering each lesson; which parts of the lesson were delivered; their view on the suitability of lesson content; and the proportion of pupils who were positively engaged in the lesson. In line with previous research on the fidelity of delivering KiVa lessons [52], the analysis will focus on adherence (to lesson content), exposure (length of lessons) and quality (using time spent preparing lessons as a proxy). Lesson adherence will be calculated as the proportion of tasks delivered for each lesson averaged over the 10 lessons (expressed as a percentage). Lesson duration will be calculated as the number of minutes used for teaching lesson content averaged across the lessons a teacher is reported to have delivered. Time spent preparing the lessons will be calculated by averaging the reported number of minutes across the lessons delivered by a teacher.

School-wide programme implementation will be assessed by independent observation (one per school, conducted in Spring 2014 for intervention schools and Spring 2015 for control schools). These school observations will be undertaken to look at how the schools are delivering the programme and examine differences and similarities in delivery across different school contexts. School-wide observations will use a list of eight items and researchers will score each one on a three-point scale (0 = “not true”, 1 = “somewhat true”, 2 = “certainly true”) with additional space for recording notes to support the score given. Items cover: the visibility of KiVa materials in the school; the extent to which the headteacher, playtime supervisors, a Key Stage 2 teacher (or the KiVa team lead) and Key Stage 2 pupils can talk fluently and knowledgably about the programme; evidence of a KiVa team logbook being used to record incidents of bullying and how they were dealt with; and whether parents know what the programme is, have received information about it from the school and have used the website (schools will select parents, and it is acknowledged that they will be unrepresentative). Scores on the school observation measure will be summed to give an overall score for each school in the range 0 to 16, where a higher score indicates stronger school-wide implementation. These scores and the observers’ comments will be used to help identify common strengths and weaknesses as regards implementation. Again, analyses will use data on KiVa delivery in intervention and waitlist control schools.

#### Scale issues

The second part of the process evaluation will explore issues relating to potentially scaling up KiVa in Wales using focus groups and structured interviews. The focus groups will be held with key stakeholders in the implementation of school-based bullying interventions, namely policy makers/anti-bullying advocates (including Welsh Government, local education authorities, anti-bullying NGOs, teacher unions), teachers and other educators (working in KiVa schools), and parents/children (also from KiVa schools). They will consist of facilitated discussion, supported by a series of questions and prompts, of three main topic areas relevant to the scalability of KiVa in Wales: the need and demand in Wales for anti-bullying programmes generally and KiVa specifically, and how to build demand; how well KiVa fits with the social, political and cultural context in Wales and with the educational context (including the curriculum); and, should the trial results be positive, how the implementation of KiVa at scale in Wales can best be enabled and supported given the context (to cover the most suitable support structure for implementation, including training, technical assistance and financing). It is planned to hold 18 focus groups, or as many as needed for data saturation (i.e., fewer may be sufficient). Focus group data will be analysed using thematic analysis [53] to identify themes relevant to developing a scalable model of the programme. Data will be coded deductively, focusing on data items related to need/demand, attitudes towards the KiVa programme, and sustainability.

The structured interviews will be conducted with headteachers in schools delivering KiVa and teachers who deliver the programme, and will elicit their experiences of implementing KiVa. The interview content will cover the following: how the programme is working in the school; what teachers like or dislike; any observed benefits for the school; any challenges of implementation (and efforts to overcome those challenges); and any reflections on how implementation may better be supported in Wales. A narrative summary of the structured interviews with teachers will be produced.

### Micro-costing

The study will estimate the cost per school and per child of setting up and implementing the KiVa programme in Wales. A micro-costing analysis will be undertaken to determine the cost of implementing KiVa in the intervention arm schools in the first year (i.e., during the trial), including set-up or non-recurrent unit costs (e.g., purchasing materials, training staff) and recurrent unit costs (e.g., staff time, yearly registration costs) for the programme. Costs related to undertaking the research evaluation will be excluded where they are not integral to the implementation of the KiVa programme. Micro-costing is a bottom-up approach used to estimate the cost of setting up and delivering an intervention. It involves collecting detailed information about the resources required to deliver an intervention, and subsequently assigning economic unit costs to each component of resource use. The alternative approach would be gross-costing, a top-down approach where the total cost invoiced is divided by the total resource use to obtain an average cost of resource use. The micro-costing approach is accepted as being more accurate than gross-costing, and is widely used in costing studies [54–56].

Costs will be presented in UK Pounds Sterling for the financial year 2013–2014. Structured forms (record books) will be developed and distributed to the designated KiVa lead at participating schools, together with the KiVa project team (responsible for providing materials, training, supervision and additional support). These record books will ascertain how much time the KiVa lead and other school staff spend each month on activities directly related to implementing KiVa (e.g., staff meetings, setting up and inviting parents to a meeting to introduce the programme). The fidelity measures will also be used to assess the amount of time teachers spend on lesson preparation and delivery. Additionally, the KiVa lead for the school will be asked to complete a structured form summarising how much time school staff spend travelling to training and supervision sessions. Time spent by teachers and other school staff at supervision sessions will be calculated using attendance records completed by the KiVa project team. The KiVa project team will also be asked to provide details summarising costs and time related to providing materials, training, supervision and additional support.

For the analysis, teacher costs will be based on data collected regarding staff time multiplied by national average salaries for a mid-point M5 qualified teacher. A school year of 38 weeks for the delivery of KiVa will be assumed, taking sickness, Continuing Professional Development and holidays into account. Salary calculations will be inclusive of employers’ on-costs (25 %). The KiVa programme costs will be separated into recurrent and non-recurrent costs and will exclude costs related to undertaking the evaluation. The various elements will be summed and divided by the number of schools (i.e., 10) delivering KiVa in the trial to give an average cost per school. In order to calculate the average cost per child, the total cost will be divided by the number of children receiving the intervention.

### Ethical approval

Ethical approval was granted by the School of Psychology, Ethical and Governance Board, at Bangor University on 30^th^ May 2013 (Ethical approval code: 2013–9162).

### Informed consent

Obtaining informed consent for this trial will be on five levels, as follows.

1. Head teachers: Subsequent to expressing their interest in the intervention, attending a recruitment meeting, and thereby being provided with the opportunity to discuss the implications of the trial, head teachers will provide written consent for their school to participate in the trial (which includes adhering to the randomisation outcome, delivering the programme in full, participating in the training and making good use of additional support). They will also consent to allow the research team to collect and use for the purposes of analysis the following: child and teacher online questionnaires (BVQ and SDQ); child-level data held on the school administrative system (ethnicity, free school meal status, SEN status, attendance); programme implementation monitoring data (online teacher lesson records completed by teachers after each KiVa lesson); school observation data; interview data; and implementation costs data.
2. Parents: It is not necessary to obtain parental consent for the intervention as the programme falls within usual curriculum and other institutional activities. However, parents of pupils in the relevant year groups in participating schools will be provided with an opt-out (passive) consent form that they must return to the respective school if they wish their child’s data (from the BVQ/SDQ) to be withdrawn from the research (they cannot withhold consent for the KiVa pupil online survey to be completed as it is part of the programme). If parents do not opt out it is assumed that they consent to the data collected on their child being used in the evaluation. (Neither children nor parents on their behalf may opt out of participating in the KiVa programme as this is being delivered as part of the PSE curriculum, which is a curriculum requirement.) Parents participating in discussions organised as part of the school observations will be required to provide active consent. As the school observations also involve a researcher asking questions of a class of children, headteachers will be encouraged to let parents know that a researcher will be visiting the school and for what purpose. It is not deemed proportional to obtain passive or active parental consent for this because the questions are non-sensitive, no information is collected about individuals per se, and the data are anonymised at source.
3. Children: Consent to take part in the programme is not required as it falls within usual curriculum and other institutional activities. However, pupils in relevant year groups will be required to provide active consent to complete the KiVa pupil online questionnaire, and will be able to stop completing the questionnaire at any time.
4. Focus groups: Headteachers, teachers, parents, children and other stakeholders will be required to provide active written consent to participating in the respective focus groups and to the information they supply being used in the research.

Bilingual (English/Welsh) consent forms will be provided in all cases.

### Project timetables and milestones

Delivery of the intervention is dictated by the school academic year (early September to mid July), so the recruitment of schools, baseline assessments and the training for intervention arm schools need to take place before the academic year 2013–14. The timetable and milestones will be set according to these criteria. Schools will be recruited in March-April 2013 and randomly allocated to intervention and control arms in May 2013. Baseline data will be collected from all schools in June/July 2013 (BVQ and SDQ). Teachers and other staff in intervention arm schools will be trained in June/July 2013. Intervention delivery will commence in intervention arm schools in September 2013. Coaching for staff in these schools will be provided in each of the three terms of the 2013–2014 academic year. Follow-up outcome data (12 months post-baseline) will be collected in all schools in June/July 2014. This follow-up will overlap with training of staff in control arm schools. Waitlist control arm schools will commence delivery of the programme in September 2014 (after the trial) and receive coaching in each of the three terms of the 2014–2015 academic year. The economic evaluation focuses on intervention arm schools only. Focus groups will take place between February and June 2014, while school observations (including interviews with school personnel) will take place in May/June 2014 for the intervention schools and May/June 2015 for the waitlist control schools. The data analysis will take place at the end of 2015.

## Discussion

This pragmatic cluster RCT will provide important information on whether the KiVa programme is transportable from Finland to Wales in terms of effectiveness and implementation. In particular, it will examine whether KiVa is effective in reducing child-reported victimisation and bullying and improving pupils’ emotional well-being and school attendance. It will provide information about the cost to deliver KiVa in Wales. In addition, it will generate insights into the need and demand for KiVa, the fit with the Welsh context and educational curriculum, and approaches to supporting the scale-up of KiVa in Wales should the findings indicate that it is effective.

## Abbreviations

BVQ: Bully/Victim Questionnaire
CAFCASS: Children and Family Court Advisory and Support Service
CEBEI: Centre for Evidence Based Early Intervention
CEIT: Children’s Early Intervention Trust
DSRU: Dartington Social Research Unit
FSM: Free school meals
ICC: Intra class correlation
ITT: Intention-to-treat
PSE: Personal and Social Education
RCT: Randomised controlled trial
SDQ: Strengths and Difficulties Questionnaire
SEN: Special educational needs

## Footnotes

1 The algorithm is tuned to allow a balance between predictability and balance between the allocated groups, within stratification variables and the strata (stratification variable combinations). As all participants will be recruited before randomisation in this instance the algorithm ensures exact balance between the groups, stratification variables and strata.

2 The score for the hyperactivity/inattention scale was 0.83 but this outcome is not examined in the present study, as it is not hypothesised that the programme will have an impact on it.

3 Data will also be collected at 24 months post-baseline (June/July 2015) because the online survey is part of the KiVa programme, but as the control group will receive the intervention in the second year of the study the data cannot be used as part of the RCT.
